# Peripheral BDNF: a candidate biomarker of healthy neural activity during learning is disrupted in schizophrenia

**DOI:** 10.1017/S0033291714001925

**Published:** 2014-08-27

**Authors:** A. J. Skilleter, C. S. Weickert, A. Vercammen, R. Lenroot, T. W. Weickert

**Affiliations:** 1 School of Psychiatry, University of New South Wales, Kensington, NSW, Australia; 2 Neuroscience Research Australia, Randwick, NSW, Australia; 3 Schizophrenia Research Institute, Darlinghurst, NSW, Australia

**Keywords:** BDNF, biomarker, brain-derived neurotrophic factor, functional MRI, plasma, probabilistic association learning, schizophrenia

## Abstract

**Background.:**

Brain-derived neurotrophic factor (BDNF) is an important regulator of synaptogenesis and synaptic plasticity underlying learning. However, a relationship between circulating BDNF levels and brain activity during learning has not been demonstrated in humans. Reduced brain BDNF levels are found in schizophrenia and functional neuroimaging studies of probabilistic association learning in schizophrenia have demonstrated reduced activity in a neural network that includes the prefrontal and parietal cortices and the caudate nucleus. We predicted that brain activity would correlate positively with peripheral BDNF levels during probabilistic association learning in healthy adults and that this relationship would be altered in schizophrenia.

**Method.:**

Twenty-five healthy adults and 17 people with schizophrenia or schizo-affective disorder performed a probabilistic association learning test during functional magnetic resonance imaging (fMRI). Plasma BDNF levels were measured by enzyme-linked immunosorbent assay (ELISA).

**Results.:**

We found a positive correlation between circulating plasma BDNF levels and brain activity in the parietal cortex in healthy adults. There was no relationship between plasma BDNF levels and task-related activity in the prefrontal, parietal or caudate regions in schizophrenia. A direct comparison of these relationships between groups revealed a significant diagnostic difference.

**Conclusions.:**

This is the first study to show a relationship between peripheral BDNF levels and cortical activity during learning, suggesting that plasma BDNF levels may reflect learning-related brain activity in healthy humans. The lack of relationship between plasma BDNF and task-related brain activity in patients suggests that circulating blood BDNF may not be indicative of learning-dependent brain activity in schizophrenia.

Schizophrenia is a severe mental disorder characterized by a diverse set of symptoms, including positive (hallucinations, delusions) and negative (lack of motivation, social withdrawal, blunted affect) symptoms and cognitive impairment. Cognitive deficits are recognized as forming part of the core psychopathology of schizophrenia, with impairments in learning, memory and attention being common (Weickert *et al.* 2000; Palmer *et al.* 2009).

Brain-derived neurotrophic factor (BDNF) is a member of the neurotrophin family of growth factors that potentiates synaptic strength and plasticity underlying learning and memory (Lu & Chow, 1999; Poo, 2001; Egan *et al.* 2003; Hariri *et al.* 2003; Lu, 2003; Dempster *et al.* 2005). A single nucleotide polymorphism in the human BDNF gene produces a valine to methionine substitution in the protein prodomain that influences intracellular trafficking, activity-dependent release of BDNF, learning and brain activity (Egan *et al.* 2003; Hariri *et al.* 2003). Lower levels of both brain and blood BDNF have also been implicated in the majority of studies of the pathophysiology of schizophrenia (Weickert *et al.* 2003; Xiu *et al.* 2009; Rizos *et al.* 2010; Green *et al.* 2011). Peripheral blood can provide an important reservoir of BDNF, reflecting BDNF secreted from the brain and BDNF that may be available to influence brain function (Schmidt & Duman, 2010). However, the extent to which circulating BDNF correlates with human brain activity during learning has not been demonstrated and the extent to which lower circulating BDNF may be indicative of the neural correlates of cognitive impairment in schizophrenia has not been shown. Low peripheral BDNF is reported in first-episode psychosis (Buckley *et al.* 2007) and is associated with cognitive impairment in chronically ill people with schizophrenia (Zhang *et al.* 2012*b*). Peripheral BDNF levels are also significantly decreased in people with mild cognitive impairment (Yu *et al.* 2008). Thus, peripheral BDNF levels may be useful as a biomarker in health and disease. However, to more fully interpret what any change in a potential biomarker may mean for brain function, a relationship between peripheral BDNF and brain activity should be established in the healthy and diseased states.

One reason that general predictions about blood–brain relationships in schizophrenia are difficult to make is that studies of peripheral BDNF levels in schizophrenia have reported inconsistent results. Although the majority of studies report decreased circulating BDNF levels (Pirildar *et al.* 2004; Tan *et al.* 2005; Grillo *et al.* 2007; Rizos *et al.* 2008), some find increased levels (Gama *et al.* 2007; Reis *et al.* 2008), and one study reports no significant difference in plasma BDNF levels in people with schizophrenia compared to healthy controls (Lee & Kim, 2009). A recent meta-analysis supports an overall reduction in peripheral BDNF levels in schizophrenia (Green *et al.* 2011). However, the extent to which alterations in blood BDNF levels relate to abnormal learning-related brain activity in schizophrenia, as compared to healthy individuals, remains to be demonstrated.

Healthy cortical neurons innervate and supply BDNF to several brain regions, including the striatum (Alexander *et al.* 1986; Altar & DiStefano, 1998). As striatal neurons are trophically dependent on BDNF supplied through anterograde transport from the frontal cortex (Hofer *et al.* 1990; Altar *et al.* 1997; Rauskolb *et al.* 2010; Dieni *et al.* 2012), higher levels of BDNF would be expected to relate to stronger frontal–striatal activity in a healthy brain, and a reduction of prefrontal cortical BDNF, as found in schizophrenia (Weickert *et al.* 2003; Hashimoto *et al.* 2005), would be expected to negatively impact striatal function in schizophrenia.

There is abundant evidence from neuropsychological and neuroimaging studies demonstrating significant learning impairments and frontal–striatal dysfunction in schizophrenia (Pantelis *et al.* 1997; Meyer-Lindenberg *et al.* 2002; Murray *et al.* 2008; Howes *et al.* 2009; Weickert *et al.* 2010; Morris *et al.* 2012). Probabilistic association learning (requiring gradual learning of probabilistic-based cue–outcome associations) elicits frontal–parietal–striatal activity in healthy people (Poldrack *et al.* 1999; Fera *et al.* 2005) and is related to reduced activity in a neural network that includes the dorsolateral prefrontal cortex (DLPFC), parietal cortex and caudate nucleus in schizophrenia (Weickert *et al.* 2009). Probabilistic association learning is impaired in people with schizophrenia (Weickert *et al.* 2002; Foerde *et al.* 2008; Horan *et al.* 2008), the offspring of people with schizophrenia who are at high risk of developing schizophrenia (Wagshal *et al.* 2012, 2013) and in unaffected siblings of people with schizophrenia (Weickert *et al.* 2010; Wagshal *et al.* 2014), with deficits in overall performance and learning rate.

In the present study, we sought to define the relationship between peripheral BDNF levels and the neural processes underlying probabilistic association learning in healthy people and people with schizophrenia. First, we confirmed the reduced brain activation in people with schizophrenia *versus* healthy controls during a probabilistic association learning test using functional magnetic resonance imaging (fMRI). Second, we tested the extent to which plasma BDNF levels are related to brain activity in healthy people during probabilistic association learning. Third, we determined whether a similar relationship was also present in schizophrenia. We predicted a positive relationship between plasma BDNF levels and brain activity in a neural network consisting of prefrontal–parietal cortices and striatum, which has previously been shown to be relevant for probabilistic association learning in healthy adults. Based on the majority of work showing dysregulation of either BDNF levels or prefrontal cortex activity along with an increase in the truncated BDNF receptor (Weickert *et al.* 2003; Hashimoto *et al.* 2005; Wong *et al.* 2010, 2013; Ray *et al.* 2014), which is thought to block trophic effects in schizophrenia, we further predicted an abnormal relationship between plasma BDNF levels and brain activity in schizophrenia.

## Method

### Participants

Twenty-eight healthy adults without a personal or family history of mental illness and 20 chronically ill adults with a diagnosis of schizophrenia or schizo-affective disorder were recruited for participation in the study. All patients met the DSM-IV criteria for schizophrenia or schizo-affective disorder on the basis of the Structured Clinical Interview for DSM-IV Axis 1 Disorders (SCID; First *et al.* 2002). All participants were screened for exclusion criteria, which included a concurrent DSM-IV Axis I diagnosis other than schizophrenia or schizo-affective disorder in patients, or any DSM-IV diagnosis in healthy adults, and for all participants: history of uncontrolled diabetes or cardiovascular disease including hypertension, recent alcohol/substance abuse (within the past 5 years), head injury with loss of consciousness, epileptic seizures, structural brain abnormalities, developmental disorders, mental retardation and/or central nervous system (CNS) infection. Healthy adults having a first-degree relative with schizophrenia or schizo-affective disorder were also excluded. A four-subtest version of the Wechsler Adult Intelligence Scale, 3rd Edition (WAIS-III; Wechsler, 1997) and the Wechsler Test of Adult Reading (WTAR; Wechsler, 2001) were administered to all participants to obtain estimates of current IQ and premorbid IQ in schizophrenia. All people with schizophrenia were receiving antipsychotic medication (82% receiving second-generation antipsychotics) for at least 1 year prior to participation and 41% were receiving antidepressant medication. All participants had normal vision or their vision was corrected to normal with MRI-compatible lenses.

Demographic characteristics of each group are shown in Table 1. The mean daily dose of antipsychotic medication for each person with schizophrenia was converted to an approximate daily mean chlorpromazine (CPZ) milligram equivalents dose using standard guidelines (Leucht *et al.* 2003; Woods, 2003). Symptom severity in people with schizophrenia was assessed by a trained psychologist or psychometrician using the Positive and Negative Syndrome Scale (PANSS; Kay *et al.* 1987). All procedures were approved by the University of New South Wales and the South Eastern Sydney and Illawarra Area Health Service Human Research Ethic Committees. The procedure was explained and written informed consent was obtained from each participant before entry into the study.

**Table 1.** Demographic summary of the schizophrenia and healthy control samples

	Schizophrenia (*n* = 17)	Controls (*n* = 25)	df	*t* value/*χ*^2^	*p*
Age (years)	38.0 (9.2)	29.2 (6.8)	40	3.57	0.001
Gender M/F	8/9	13/12	1	0.10	0.750
Education years	14.4 (2.7)	15.1 (2.0)	40	0.99	0.330
WAIS-III FSIQ	95.5 (11.9)	107.8 (12.1)	40	3.26	0.002
WTAR	104.8 (4.9)	110.2 (6.0)	40	3.00	0.004
Schizophrenia/schizo-affective disorder	8/9				
Age of illness onset (years)	25.2 (7.2)				
Duration of illness (years)	12.9 (6.6)				
CPZ equivalence dose (mg/day)	552.8 (416.3)				
PANSS Positive	13.3 (5.1)				
PANSS Negative	14.0 (4.9)				
Antipsychotic medications	Amisulpride 2				
	Clozapine + aripiprazole 1				
	Clozapine + chlorpromazine 1				
	Clozapine + haloperidol 1				
	Clozapine + paliperidone 1				
	Clozapine + risperidone 2				
	Flupentixol 1				
	Haloperidol 1				
	Olanzapine 1				
	Quetiapine 2				
	Quetiapine + zuclopenthixol 1				
	Ziprasidone 2				
	Zuclopenthixol 1				
Antidepressant medications	Citalopram 1				
	Escitalopram 1				
	Escitaolpram + lithium carbonate 1				
	Fluoxetine 1				
	Lithium 1				
	Sertraline 1				
	Venlafaxine 1				
Other medications	Albuterol 1				
	Atorvastatin 2				
	Benztropine 1				
	Calcium carbonate 1				
	Diazepam 1				
	Metformin 2				
	Metoprolol 1				
	Pantoprazole 1				
	Sodium valproate 3				
	Telmisartan 1				
	Thyroxine 1				

M, Male; F, female; WAIS-III FSIQ, Wechsler Adult Intelligence Scale 3rd edition full-scale IQ estimate; WTAR, Wechsler Test of Adult Reading; CPZ, chlorpromazine; PANSS, Positive and Negative Syndrome Scale; df, degrees of freedom.

Means or numbers provided. Standard deviation in parentheses.

### Circulating BDNF level assay

Plasma samples from all participants were collected in 9-ml ethylenediaminetetraacetic acid (EDTA) tubes between 0900 and 1100 h on the day of the MRI scan, put on ice and stored at −80°C until the day of assay. BDNF protein was measured in each plasma sample by an enzyme-linked immunosorbent assay (ELISA) kit (no. G7611, Promega Corporation, USA) according to standard protocol (see online Supplementary Material for details). All samples were assayed in duplicate in each plate over two different assay days (with up to four measurements for each participant).

### Probabilistic association learning Weather Prediction Task (WPT)

Participants completed a probabilistic association learning WPT in the scanner, which alternates the experimental and perceptual-motor control tests as described in detail previously (Poldrack *et al.* 1999; Fera *et al.* 2005; Weickert *et al.* 2009) (see online Supplementary Material).

### Scanning procedure

A 3-T Phillips Achieva MRI scanner with an eight-channel birdcage head coil at Neuroscience Research Australia, Randwick, Australia was used to acquire 162 whole-brain echo planar images: repetition time/echo time (TR/TE) = 3000/30, 45 interleaved slices, thickness = 3 mm, gap = 0.3 mm, voxel size = 2.14 × 2.14 × 3 mm, flip angle = 90°, field of view (FOV) = 240 mm. A T1-weighted high-resolution anatomical scan was obtained for each participant for registration purposes: TR/TE = 5.3/2.4, 180 slices, thickness = 1 mm, no gap, voxel size = 1 × 1 × 1 mm, FOV = 256 mm.

The experimental paradigm consisted of 16 blocks of 30 s each in which weather prediction blocks (six trials/block) alternated with the control task (six trials/block). There were 48 weather prediction and 48 perceptual-motor control trials in total, equating to eight blocks of each condition. One hundred and sixty-two scans were collected in a total scan time of 8.25 min. Stimuli for both tests were presented on an inverted computer screen that participants could see using a mirror mounted in the MRI head coil. Behavioral responses (left button press for ‘rain/two’ and right button press for ‘shine/not two’) were made with the right thumb and were recorded using a fiber-optic response box (Lumina Systems, Cedrus Corp., USA), which collected accuracy (number correct) and reaction time (ms) measures. All stimuli were displayed on the screen for 4.5 s with an inter-trial interval of 0.5 s. The words ‘correct’ or ‘incorrect’ appeared immediately following a button press response as feedback to the participant during both the weather prediction and perceptual-motor control tests. Missed trials were not included in the scoring.

### fMRI processing

Preprocessing was performed with SPM8 (Wellcome Trust Centre for Neuroimaging, UK), running under MATLAB version 2010b. Functional images were realigned to the first image in the sequence. Three dummy scans were obtained before each fMRI data acquisition to allow for equilibration of the MRI signal. Functional images were co-registered with the anatomical image. Images were smoothed with an 8-mm full-width at half-maximum (FWHM) Gaussian kernel. Movement parameters created during pre-processing were applied as a regressor covariate. Anatomical scans were also screened for structural abnormalities by a radiologist. All data sets were screened for artifacts, excessive movement (>3 mm along *x, y* or *z* axes), and unsuccessful normalization. We excluded three healthy controls and three people with schizophrenia due to excessive movement, leaving 25 controls and 17 people with schizophrenia whose data were entered into our analyses.

### Data analysis

#### Group comparisons on demographics and BDNF

Demographic variables and plasma BDNF levels of the schizophrenia and healthy control groups were compared using *t* tests and ANOVAs for continuous variables or *χ*^2^ tests for categorical variables. Although the groups differed significantly on age (*p* = 0.001), with controls significantly younger than people with schizophrenia (see Results), our main objective was to compare blood BDNF and blood oxygen level-dependent (BOLD) activity within diagnostic groups and not to test for a diagnostic difference between groups. Thus, we opted to retain the largest number of participants as possible rather than match on the basis of age. The degree of correlation among plasma BDNF levels, age, PANSS scores and CPZ equivalent dose was performed using Pearson's product-moment correlations. Statistica version 12 was used for statistical analyses of behavioral and clinical measures. The *α* level for statistical significance was set at *p* < 0.05.

#### Probabilistic association learning analysis

The mean cumulative percentage correct after 48 trials was used as a measure of learning the cue–outcome associations. We also performed separate *t* tests at each trial block relative to chance (50% correct) in each group to test for significant improvement from chance levels.

#### fMRI analyses

A description of the whole-brain analysis is presented in the online Supplementary Material.

#### Region of interest (ROI) regression analysis of the relationship of BDNF on frontal–parietal–striatal activity

The novel analysis in this study was an ROI analysis in which the ROIs were chosen *a priori* based on the frontal–parietal–striatal brain regions previously related to probabilistic association learning in healthy adults (Poldrack *et al.* 1999; Fera *et al.* 2005; Weickert *et al.* 2009). Bilateral DLPFC, parietal cortex and caudate nucleus ROIs were defined structurally according to the Anatomical Automatic Labeling atlas (Tzourio-Mazoyer *et al.* 2002), such that there were six ROIs in total and *β* weights were extracted from each ROI. Plasma BDNF levels were then correlated with the *β* weights from each ROI within each group and a family-wise error (FWE) rate correction for multiple comparisons was applied. Correlation strengths between groups were compared directly using a Fisher *r* to *z* transformation. Correlation of daily CPZ equivalent dose and *β* weights from each ROI were also performed in people with schizophrenia to determine the extent to which antipsychotics influence brain activation during learning.

## Ethical considerations

All procedures contributing to this work complied with the ethical standards of the relevant national and institutional committees on human experimentation and with the Helsinki Declaration of 1975, as revised in 2008.

## Results

### Demographic measures

The results of the demographic analyses are provided in Table 1. The groups did not differ significantly on the basis of gender distributions or education. There were expected significant group differences on WAIS-III IQ and WTAR scores, such that people with schizophrenia scored significantly lower than controls.

### Probabilistic association learning and plasma BDNF levels

Plasma BDNF levels and probabilistic association learning performance of participants during the scan are presented in Table 2. Plasma BDNF levels were significantly increased in schizophrenia relative to controls. An ANOVA with sex and diagnostic status as grouping factors and BDNF levels as the dependent variable showed a main significant effect of diagnostic group (*F*_1,38_ = 6.01, *p* = 0.02), such that controls had lower plasma BDNF levels than people with schizophrenia. There was no significant main effect of sex on plasma BDNF levels (*F*_1,38_ = 3.44, *p* = 0.07). There was a significant interaction between sex and diagnostic group on plasma BDNF levels (*F*_1,38_ = 6.32, *p* = 0.02), such that there was no significant difference in plasma BDNF levels on the basis of sex in the control group (*p* = 0.61); however, there were significant differences in plasma BDNF levels on the basis of sex in the patient group, in which the female patients displayed significantly higher plasma BDNF levels than male patients (*p* = 0.007) and both male (*p* = 0.003) and female healthy controls (*p* = 0.001).

**Table 2.** Mean plasma BDNF levels and percentage correct during probabilistic association learning in people with schizophrenia and healthy controls

	Schizophrenia	Controls	*t* value	df	*p*
Plasma BDNF (ng/ml)	1.83 (1.49)	1.00 (0.75)	−2.39	40	0.02
Probabilistic association learning percentage correct over 48 trials	61.2 (9.0)	65.4 (6.7)	1.7	40	0.09
Number of missed trials	0.5 (0.8)	0.4 (0.8)	−0.2	40	0.83

BDNF, Brain-derived neurotrophic factor; df, degrees of freedom.

Standard deviation in parentheses.

The results of an unmatched *t* test on the cumulative mean percentage correct after 48 trials of probabilistic association learning revealed a trend towards a significant group difference, such that controls tended to perform better than people with schizophrenia. Regarding performance relative to chance, controls performed significantly above chance levels from block 2 to block 8 (*p* < 0.001) and people with schizophrenia performed significantly above chance levels from block 4 to block 8 (*p* < 0.01). Both groups missed less than 1% of the total number of trials and there was no significant difference between groups with respect to the mean number of missed trials (see Table 2).

No strong, significant correlations were obtained between plasma BDNF levels and probabilistic association learning performance after 48 trials in controls (*r* = 0.19, *p* = 0.36) or in people with schizophrenia (*r* = 0.17, *p* = 0.53). There was a non-significant positive correlation between plasma BDNF level and daily CPZ equivalent dose in people with schizophrenia (*r* = 0.36, *p* = 0.16).

### fMRI analysis results

**Fig. 1.**
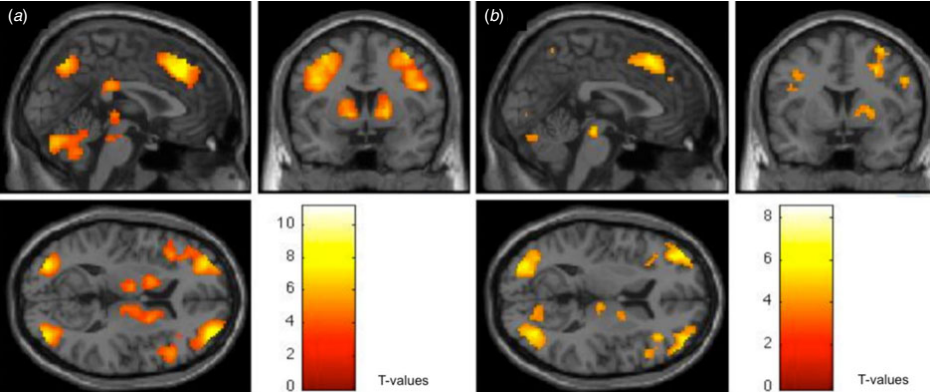
Dorsolateral prefrontal cortex (DLPFC), parietal cortex and caudate nucleus activity in (*a*) healthy adults and (*b*) people with schizophrenia during probabilistic association learning [*p* = 0.01, false discovery rate (FDR) corrected].

Healthy controls showed increased activation in frontal–striatal and parietal regions during probabilistic association learning (Fig. 1*a* and online Supplementary Table S2). People with schizophrenia also showed activity in similar frontal–striatal and parietal regions during probabilistic association learning, although this seemed to be less robust (Fig. 1*b* and online Supplementary Table S2).

**Fig. 2.**
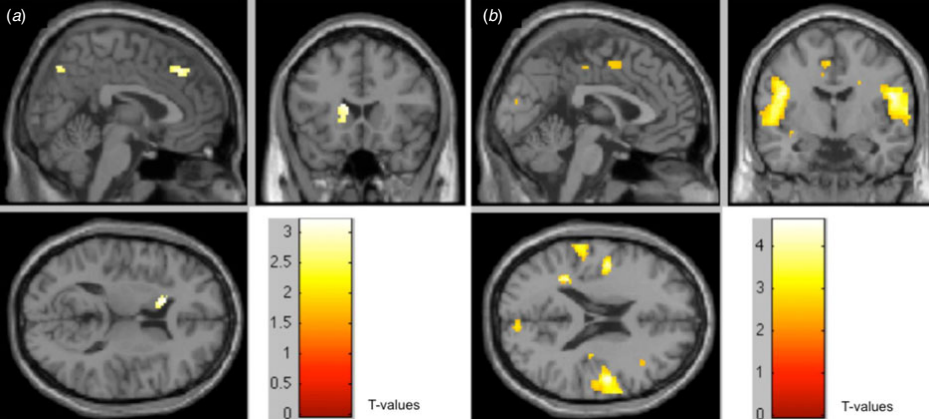
Direct comparisons between the two groups show areas where task-related activity is (*a*) greater in healthy controls than in people with schizophrenia and (*b*) greater in people with schizophrenia than healthy controls (*p* = 0.0005).

Direct comparisons between groups revealed that the medial frontal gyri, right precuneus and left caudate were more active in healthy controls during probabilistic association learning compared to people with schizophrenia (Fig. 2*a*), replicating our previous work (Weickert *et al.* 2009). A larger network encompassing the temporal lobes, inferior frontal gyrus, cingulate gyrus and postcentral gyrus was found to show greater activity in people with schizophrenia compared to healthy controls (Fig. 2*b*), also replicating our previous findings.

### ROI analyses: correlations with plasma BDNF levels

**Fig. 3.**
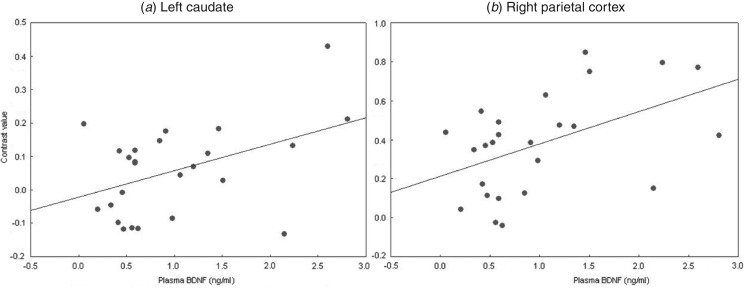
Correlation between plasma brain-derived neurotrophic factor (BDNF) and brain activity in (*a*) the left caudate and (*b*) the right parietal cortex of healthy controls.

Plasma BDNF levels showed strong, significant, positive correlations with brain activation in the bilateral caudate and right parietal cortex in healthy adults during probabilistic association learning (Table 3 and Fig. 3). After applying FWE rate corrections for multiple comparisons using Hochberg's step-up procedure, only the correlation between plasma BDNF levels and activity in the right parietal cortex remained significant. There were no strong, significant correlations between plasma BDNF levels and brain activation in the frontal cortex during learning in healthy adults. There were no strong, significant correlations between plasma BDNF levels and activation in any of the six ROIs in people with schizophrenia (Table 3). The results of the Fisher *r* to *z* transformation revealed that the correlation between the right parietal cortex and peripheral BDNF levels was significantly different between healthy controls and people with schizophrenia (Table 4) and the correlation of BDNF with the left caudate nucleus showed a trend towards a significant difference between groups (*p* = 0.06).

**Table 3.** Correlations between plasma BDNF levels and brain activity during probabilistic association learning in people with schizophrenia and healthy controls

	Left DLPFC	Right DLPFC	Left caudate	Right caudate	Left parietal cortex	Right parietal cortex
Schizophrenia	*r*=−0.05	*r* = 0.13	*r* = − 0.07	*r* = 0.07	*r* = 0.04	*r* = − 0.03
	*p* = 0.85	*p* = 0.63	*p* = 0.78	*p* = 0.78	*p* = 0.88	*p* = 0.92
Controls	*r* = 0.08	*r* = 0.16	*r* = 0.44	*r* = 0.40	*r* = 0.27	*r* = 0.49
	*p* = 0.71	*p* = 0.45	*p* = 0.03	*p* = 0.05	*p* = 0.20	*p* = 0.01*

BDNF, Brain-derived neurotrophic factor; DLPFC, dorsolateral prefrontal cortex.

* Significantly different after family-wise error (FWE) rate correction for multiple comparisons.

**Table 4.** Fisher's r to z transformation comparing correlation strengths of BDNF levels and brain activity between people with schizophrenia and healthy controls

	Left DLPFC	Right DLPFC	Left caudate	Right caudate	Left parietal cortex	Right parietal cortex
Z	−0.38	−0.09	−1.59	−1.03	−0.69	−1.66
*p* value	0.35	0.46	0.06	0.15	0.25	0.05

BDNF, Brain-derived neurotrophic factor; DLPFC, dorsolateral prefrontal cortex.

### Correlations of BDNF levels and brain activity with other variables

There were no strong, significant correlations among plasma BDNF levels and the following variables in patients and/or controls: brain activity in the ROIs for the motor control task, age and symptom severity (in patients) (see online Supplementary Tables S3–S5). There were also no strong, significant correlations among brain activity in the ROIs examined and the following factors in patients: daily CPZ equivalent dose and symptom severity (see online Supplementary Tables S6–S7).

## Discussion

This is the first study to report a significant positive relationship between plasma BDNF levels and brain activity in the right parietal cortex, which receives prominent afferent input from the frontal cortex, during probabilistic association learning in healthy adults. Additionally, supporting our prediction of an altered relationship between plasma BDNF and brain activity in schizophrenia, people with schizophrenia showed no relationship between plasma BDNF and neural activity in the frontal–parietal–striatal network. We found that the relationship between plasma BDNF and parietal cortex activity was significantly different between groups, with a trend towards a group difference in the caudate. Indeed, in people with schizophrenia, as plasma BDNF levels increased, no parallel change in task-related brain activity was detected in the cortical or subcortical regions examined.

Previous studies have also suggested that circulating BDNF may be a biomarker of memory and of general cognitive function in healthy adults, supporting the hypothesis that it could be a surrogate marker of cortical function in humans (Gunstad *et al.* 2008; Komulainen *et al.* 2008). Our finding provides the first biological link between peripheral brain-derived growth factors and brain physiology in healthy humans. However, we did not identify positive correlations between plasma BDNF and prefrontal cortex activity, one of the main regions of interest in our study where task-related activity is high and where BDNF synthesis is high. This was surprising because cortical activity is one of the strongest inducers of BDNF levels in the brain (Gall *et al.* 1991; Dugich-Djordjevic *et al.* 1992; Wetmore *et al.* 1994) and BDNF is thought to act locally to increase synaptic strength. However, BDNF is made in highest abundance in pyramidal neurons in cortical layer VI, which project out of the local cortical area to subcortical and other cortical regions (Koralek *et al.* 1990; Fabri & Burton, 1991). Furthermore, *in vivo* studies (Rauskolb *et al.* 2010) suggest that the major mode of BDNF action is through anterograde supply to target regions, which would be consistent with frontally derived BDNF being more strongly linked to parietal cortex (and possibly caudate) activity, as we find in our present study. Thus, we speculate that increased activity of the DLPFC may have equal or greater impact on areas poised to receive BDNF from the DLPFC rather than within the DLPFC.

The observed relationship between blood BDNF levels and brain activity illustrates the role of blood BDNF in the healthy brain; however, the significantly different correlation between plasma BDNF levels and task-related neural activity in schizophrenia suggests that blood BDNF may not be a suitable biomarker of functional or task-related activity in schizophrenia. There may be different reasons for the absence of a linear relationship between blood BDNF and brain activity in schizophrenia. As cortical activity is one of the strongest inducers of BDNF levels in the brain (Gall *et al.* 1991; Dugich-Djordjevic *et al.* 1992; Wetmore *et al.* 1994) and cortical activity is often reduced in schizophrenia (Berman *et al.* 1986; Vercammen *et al.* 2012), then decreased activity in schizophrenia should lead to reduced BDNF levels. Several studies have shown a reduction of BDNF mRNA, protein and BDNF trkB receptor in schizophrenia (Iritani *et al.* 2003; Weickert *et al.* 2003; Hashimoto *et al.* 2005; Wong *et al.* 2010; Ray *et al.* 2014). Additionally, compromised axonal transport of BDNF from the cortex may yield less bioavailable BDNF within the striatum in schizophrenia. Thus, significantly reduced cortical BDNF levels in schizophrenia (representing a floor effect) might explain why there was no correlation with neural activity in the ROIs examined.

Conversely, we found that plasma BDNF levels were significantly increased in our cohort of people with schizophrenia compared to healthy adults. This suggests that there may be increased synthesis of BDNF (and availability in circulation) in our patient sample; thus, reduced BDNF may not be the most likely explanation for a loss of frontal–striatal activity in our study. However, it is possible that the BDNF may not be readily available to the neural regions typically activated during probabilistic association learning in schizophrenia leading to an uncoupling of blood BDNF levels and brain activity. In support of this possibility, we have found that the normal function of BDNF may be interrupted due to increased truncated trkB receptors in brains of people with schizophrenia (Wong *et al.* 2013). Lower levels of the full-length BDNF receptor have also been reported in cortical and hippocampal regions in schizophrenia (Weickert *et al.* 2005; Thompson Ray *et al.* 2011; Ray *et al.* 2014). We suggest that although most people with schizophrenia may synthesize less cortical BDNF, even when BDNF levels are normal or increased, there still may be a block in the function of BDNF through increased presence of truncated trkB receptors within the brains of people with schizophrenia.

Antipsychotics may also contribute to the abnormal relationship between plasma BDNF levels and caudate activity in schizophrenia. In the present study, there was a moderately strong, but non-significant, trend towards a positive relationship between daily antipsychotic dose and plasma BDNF levels, such that as the antipsychotic dose increased, circulating BDNF levels also increased, which supports a recent study showing a positive relationship between BDNF levels and antipsychotic dose (Pedrini *et al.* 2011). Antipsychotics tend to decrease dopamine binding in the caudate nucleus in schizophrenia (Kegeles *et al.* 2008), which may result in changes to caudate activity. It is thus possible that antipsychotics may be responsible for both the increased BDNF levels in our cohort of people with schizophrenia and the blunted caudate nucleus activity seen during probabilistic association learning. However, the literature to date in this area is inconsistent and other studies of antipsychotic effects on BDNF levels report that both peripheral (Tan *et al.* 2005; Grillo *et al.* 2007; Rizos *et al.* 2010) and brain BDNF levels (Lipska *et al.* 2001; Chlan-Fourney *et al.* 2002) are often down-regulated by antipsychotics.

There are several potential limitations to the present study. It is possible that the lack of a BOLD–BDNF relationship in people with schizophrenia may be due to potential confounds or epiphenomena in a relatively small sample. However, our sample size is consistent with other fMRI studies of people with schizophrenia and, based on the between-groups statistical comparison, we were able to demonstrate a significant difference between groups.

The relationship between plasma BDNF levels and BDNF levels in the brain is not well established in humans; BDNF is expressed at relatively high levels in peripheral tissues, including the lung, heart and bone marrow (Scarisbrick *et al.* 1993; Yamamoto *et al.* 1996; Takemura *et al.* 2012), with T cells being a cellular source of BDNF (Braun *et al.* 1999). BDNF is also found in tissue that supports sensory nerve innervation (e.g. muscle and skin) and increased BDNF from target tissues can increase innervation from sensory neurons (Ernfors *et al.* 1994; LeMaster *et al.* 1999; Hsieh *et al.* 2013). Thus, it is possible that the source of BDNF in our study is peripheral and is not derived from the CNS. In addition, much of the circulating BDNF is stored in platelets, which is released upon clotting. However, it is less likely that the peripheral BDNF assayed in our study is platelet derived because we assayed plasma samples in which there is no clotting. Thus, we speculate that the plasma BDNF may be derived at least in part from the CNS. Indeed, some evidence has accrued from animal studies to suggest that there is a relationship between blood and brain BDNF levels. These studies have shown a relationship between measures of blood and brain BDNF levels (Karege *et al.* 2002) that are increased by neural activity and specifically glutamatergic synaptic activity (Goodman *et al.* 1996; Hartmann *et al.* 2001). These animal studies have shown that BDNF from peripheral blood can come from the brain and that BDNF does cross the blood–brain barrier (Pan *et al.* 1998; Sartorius *et al.* 2009) whereas one study has shown a change in BDNF with age that was not found in platelets (Karege *et al.* 2002). Thus, there is evidence from animal studies to suggest that the BDNF we measured in peripheral human blood does not necessarily come from the platelets but instead may come from the brain and specifically glutamatergic synapse activity.

Another limitation pertains to the lack of a relationship between plasma BDNF levels and probabilistic association learning performance in either group despite positive correlations with brain activity. One possible explanation may be the restricted range of probabilistic association learning performance in both groups, which has been previously reported in the WPT (Weickert *et al.* 2002). Lack of a relationship between plasma BDNF levels and probabilistic association learning should not be due to a lack of learning in the groups on the WPT because healthy controls showed performance above chance levels from trial block 2 onwards and patients showed performance above chance levels from trial block 4 onwards.

There was a significant difference in age between the groups, which may influence BDNF levels, brain activity and learning. We obtained a significant relationship between age and BOLD activity only in the right caudate nucleus in controls. We did not obtain significant group differences in learning even though the groups differed significantly in age. We recruited from a relatively restricted age range (18–50 years) to reduce the effects of age. Correlations between plasma BDNF levels and age showed no strong, significant relationship between BDNF and age in this sample, which may be due to the relatively restricted age range in our sample. Increasing age is generally associated with decreasing BDNF levels (Lommatzsch *et al.* 2005). Conversely, we obtained an effect that was opposite to what would be predicted, such that patients with schizophrenia (who were significantly older than the controls) displayed significantly higher BDNF levels than controls. Another factor, such as sex, may be responsible for the increased BDNF levels in the patients. We showed that females with schizophrenia displayed increased plasma BDNF levels relative to males with schizophrenia and healthy men and women. At 53% women with schizophrenia, our sample had a higher prevalence of women than most studies of schizophrenia. However, our sample sizes of men and women with schizophrenia were small, and hence this effect would need to be replicated in larger samples. Some studies have shown increased peripheral BDNF levels in females relative to men (Zhang *et al.* 2012*a*).

The difference between our findings and the majority of other studies on peripheral BDNF in chronically ill people with schizophrenia may be explained by the fact that our patient sample contained some recent-onset patients. One of the most potent inducers of brain BDNF is cortical glutamate levels and a recent meta-analysis suggests that cortical glutamate levels may be higher in the early phase of illness (Marsman *et al.* 2013). As our patient sample included some individuals who were within the first 5 years of diagnosis, they may have been at a higher glutamate state overall, relative to a more chronically ill sample when cortical glutamate has been consistently shown to be decreased (Marsman *et al.* 2013). Furthermore, many of the people with schizophrenia in our study had a diagnosis of schizo-affective disorder and were receiving clozapine and/or antidepressants, which may increase BDNF levels (Pedrini *et al.* 2011; Thompson Ray *et al.* 2011; Kim *et al.* 2012; Martocchia *et al.* 2014; Mitic *et al.* 2014; Ray *et al.* 2014).

We did not exclude smokers in our sample. In our study, the effects of nicotine were not controlled. As is often the case in comparisons of people with schizophrenia to control groups, there was a higher prevalence of smokers in the patient group. There would have been no nicotine withdrawal effects because smokers were allowed to smoke *ad libitum*. A recent study (Paelecke-Habermann *et al.* 2013) has also shown no significant difference in probabilistic association learning between satiated and abstinent smokers; however, there was a significant difference in probabilistic association learning between non-smokers and both casual and chronic smokers, in which the smokers displayed impaired learning. Although the prevalence of smokers was greater in the people with schizophrenia in our study, there were no significant differences in learning between the patients and healthy controls.

In summary, the findings from the present study show the first positive relationship between plasma BDNF levels and parietal cortex (and possibly caudate) activity in healthy adults during probabilistic association learning, but no relationship between plasma BDNF levels and brain activity in schizophrenia. These findings suggest that plasma BDNF levels may reflect cortical BDNF signaling during learning in healthy adults. Conversely, in schizophrenia, peripheral BDNF levels do not relate to prefrontal–parietal cortex and caudate nucleus activity, providing further evidence of abnormal BDNF trophic activity and blunted frontal–parietal–striatal function (Pantelis *et al.* 1997; Meyer-Lindenberg *et al.* 2002; Weickert *et al.* 2009). Future studies to determine the mechanism of the abnormal BDNF and brain activity relationship in schizophrenia would provide further insight into the role of trophic factors in schizophrenia. This preliminary study is the first to describe a relationship between plasma BDNF levels and neural activity during learning in healthy adults, which is disrupted in schizophrenia.

## Supplementary material

For supplementary material accompanying this paper visit http://dx.doi.org/10.1017/S0033291714001925.
